# The Dynamics of Naturally Acquired Immunity to *Plasmodium falciparum* Infection

**DOI:** 10.1371/journal.pcbi.1002729

**Published:** 2012-10-18

**Authors:** Mykola Pinkevych, Janka Petravic, Kiprotich Chelimo, James W. Kazura, Ann M. Moormann, Miles P. Davenport

**Affiliations:** 1 Centre for Vascular Research, University of New South Wales, Sydney, New South Wales, Australia; 2 Kenya Medical Research Institute, Kisumu, Kenya; 3 Case Western Reserve University, Cleveland, Ohio, United States of America; 4 University of Massachusetts Medical School, Worcester, Massachusetts, United States of America; Utrecht University, Netherlands

## Abstract

Severe malaria occurs predominantly in young children and immunity to clinical disease is associated with cumulative exposure in holoendemic settings. The relative contribution of immunity against various stages of the parasite life cycle that results in controlling infection and limiting disease is not well understood. Here we analyse the dynamics of Plasmodium falciparum malaria infection after treatment in a cohort of 197 healthy study participants of different ages in order to model naturally acquired immunity. We find that both delayed time-to-infection and reductions in asymptomatic parasitaemias in older age groups can be explained by immunity that reduces the growth of blood stage as opposed to liver stage parasites. We found that this mechanism would require at least two components – a rapidly acting strain-specific component, as well as a slowly acquired cross-reactive or general immunity to all strains. Analysis and modelling of malaria infection dynamics and naturally acquired immunity with age provides important insights into what mechanisms of immune control may be harnessed by malaria vaccine strategists.

## Author Summary

Human malaria infections resulting in serious complications and death occur predominantly in young children, and resistance is gradually acquired with repeated exposure. Malaria parasites have two major stages within the human host during its life cycle: an initial liver stage, and the subsequent blood stage, where parasites replicate in and destroy red blood cells. The mechanisms of acquired resistance to severe malaria may involve immunity to both the liver and blood stage parasites. However the relative contribution of each type of immunity is not yet understood. To gain novel insight, we have analysed data from a malaria exposed cohort from western Kenya. We used mathematical modeling to understand what form of immunity is consistent with the observed rates of reinfection in adults and children seen in the field study data. We found that the reinfection pattern can be completely explained by blood stage immunity. Moreover, the blood stage immunity must consist of rapidly-induced strain-specific immunity that clears individual infections, and general immunity that accumulates slowly and decreases the average parasite growth rate with age. Understanding the dynamics of naturally acquired immunity and infection provides important insights for effective vaccine development.

## Introduction

*Plasmodium falciparum* (Pf) malaria in holoendemic areas is characterized by high level parasitaemia and symptomatic infections in early childhood, followed by the development of semi-protective immunity that allows the persistence of low level asymptomatic infections and appears to reduce the likelihood of becoming infected if bitten by an infective mosquito. The mechanisms mediating anti-infection and anti-disease immunity are complex but are thought to include innate and adaptive immune responses that limit both the liver and blood stage of the parasite life cycle in the human host [1], [2].

One approach to understanding acquired immunity to Pf-malaria has been to study correlates of protection by measuring point-prevalence levels of immunity and prospectively assessing the infection status and clinical disease. A number of studies have elaborated upon this approach by first treating patients with anti-malarial drugs to eliminate blood-stage malaria infection, and then observing the time to natural (re)-infection in an endemic setting [3]. By measuring immune parameters at baseline, and observing their association with time-to-infection, it may be possible to identify the immune responses most important for protection from malaria. A significant difficulty with these studies is that most immune responses to malaria increase with age and after cumulative exposure to malaria antigens, and so it is often unclear whether measured responses actually mediate protection or are merely a historical marker of past exposure [4], [5]. Both antibody specificity and isotype may play a role in protection [6], [7] and indeed the specific assay used to measure immune function can lead to contradictory or inconsistent conclusions. For example, antibodies that are reactive in an ELISA assay tend to increase with age, but are often not correlated with protection when corrected for age [8]. Antibodies detected using a functional assay that measures inhibition of parasite growth show an increase with age, and an association with protection from clinical disease, but not from infection [9]. Surprisingly, growth inhibitory antibodies (that can restrict parasite growth *in vitro*) are associated with a delay in time-to-infection for individuals within a given age group, but the level of inhibition decreases with age [3], [10]. Independent of these experimental studies, modeling of malaria infection has also attempted to understand the possible cross-reactivity and molecular targets of malaria immunity, using a heuristic approach based on a qualitative assessment of the data

An alternative approach to understanding anti-malarial immunity is to study the dynamics of infection and then predict how these dynamics would be influenced by acquired immunity. That is, by comparing the infection dynamics observed in susceptible (children) in contrast to resistant (adult) populations, we can use a reverse-engineering approach to understand the differences observed in infection and growth, and predict what immune mechanisms are compatible with such an outcome. Here we use such a modeling approach to understand the effects of naturally acquired immunity on the dynamics of *P. falciparum* malaria infection in a cohort of 197 people from a holoendemic region of western Kenya.

## Materials and Methods

### A treatment time-to-infection cohort study

**Figure 1:**
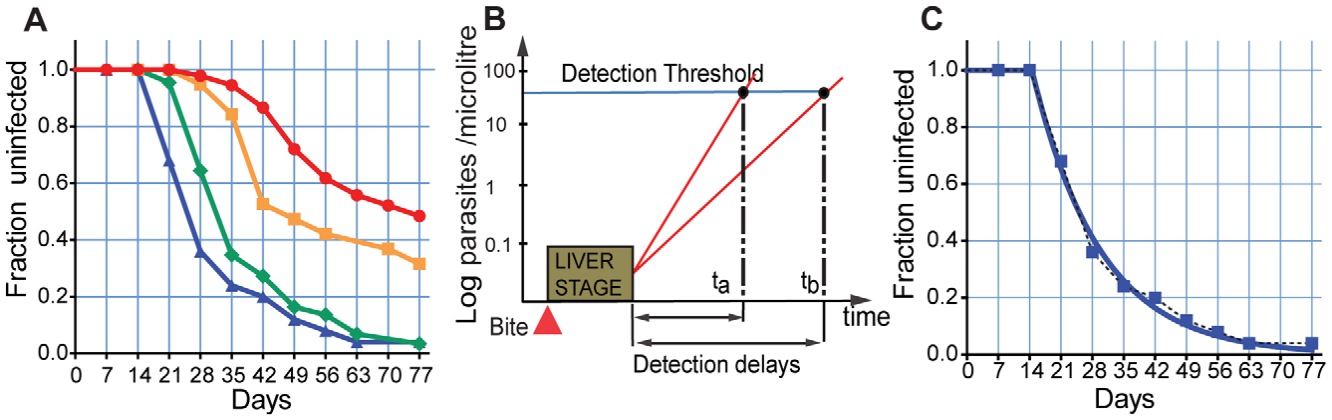
The dynamics of reinfection. Panel A. The rates of reinfection in the clinical cohort are shown for each age group over time. The proportion uninfected is shown as a ‘survival curve’ for each age group. Patients were censored if they had missing values or were treated prior to reinfection. Blue triangle and blue line - C1, green diamond and green line - C2, orange square and orange line - C3, red circle and red line - A. Panel B. Schematic illustration of parasite growth in blood. Infection commences with an infected bite, followed by a liver stage of one week. Once the blood stage commences, rapid parasite growth (high PMR) will lead to early detection on infection. Factors that slow parasite growth in the blood will delay the time until detection. Panel C. Exponential fit to field study data for group C1.

The details of cohort study have been described elsewhere [3]. Briefly, upon entry into the study (day 0) study participants (n = 201) were treated with Coartem®, which is expected to eradicate blood stage infection but is not effective against liver stage parasites [11]. Therefore, if a study participant was parasitaemic at week two post-treatment, this was considered an emerging liver stage infection and they were eliminated from further study. After treatment, blood smears were monitored weekly for 11 weeks by examination of thick and thin blood smears using light microscopy for the presence of *Pf*-malaria parasites. In addition, if weekly samples were not collected after week two post-treatment, then the study participant was eliminated; thus leaving 197 remaining for analysis. The cohort was divided into four age groups based on the immuno-epidemiology of malaria; C1 (children 1–4 years old (y.o.) were the least likely to have developed anti-malarial immunity and thus most susceptible to rapid infection; C2 (5–9 y.o.) have begun to develop partially protective immunity though may have limited strain-specific immunity; C3 (10–14 y.o.) have developed anti-malarial immunity that should begin to contain a broader repertoire of strain-specific immunity due to cumulative exposure; and A (adults>15 y.o.) who have developed anti-malarial immunity that decreases the rate of infection, parasite growth and provides coverage across many strains. Fig. 1A shows the infection curves for each age group over the 11 weeks of the study.

### The dynamics of infection

The early stages of infection involve an infected bite, infection of a liver cell, and maturation of the liver stage parasite over 6–7 days [12]. After this, the infected liver cells rupture, releasing approximately 20,000 merozoites [13]. These merozoites then initiate the blood stage infection, and successive rounds of blood stage replication follow, each lasting approximately 2 days. These earliest events during human liver and early blood stage infection are not measured in our cohort, and we first detect parasites when their density in blood reaches our detection threshold (∼40 parasites/µl). Both the rate of initiation of blood stage infection as well as the rate of growth of blood stage immunity will affect the time to first detection of infection (Fig. 1B). Liver stage immunity acts to block some infected bites from reaching the blood stage, and thus the initiation of the blood stage infection will occur with a frequency less than or equal to the real infected biting rate, and occur approximately seven days after the infected bite. Once blood stage infection is initiated, parasites grow at a rate determined by how many merozoites successfully invade new RBCs, and grow to maturity over the two-day life-cycle. We might consider this the ‘parasite multiplication rate’ (PMR), which reflects the fold increase in parasitaemia over the two day life cycle of an infected RBC. Thus, the concentration (*C*) of parasites during their growth in the blood stage can be described by formula
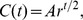
where *A* is the initial concentration of parasitized RBC in blood produced by the liver stage (estimated elsewhere [13]), *r* is the PMR and *t* is the time in days passed after initiation of blood stage. Incorporating the rate of initiation of blood stage infection (*k*), we expect the proportion of uninfected individuals remaining at a given time (*S*(*t*)) to follow the equation:
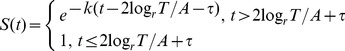
(1)where *T* is the detection threshold and *τ* is the first possible moment of blood stage infection after treatment.

In our cohort, the youngest children are the most-susceptible to infection (have the least immunity), and thus we use this group as a baseline from which to observe the effects of age-acquired immunity in the older cohorts. The infection curve of the youngest children seems to conform well with the simple dynamics described in equation (1) (Fig. 1.C). Thus, assuming that the earliest initiation of the blood stage (*τ*) is day 7 (due to the pharmacodynamics of lumafantrine), we found the rate of initiation of blood stage infection (*k*) at 0.066/day, with 95% confidence interval (CI) of (0.056, 0.076), and PMR (*r*) of 5.9, CI (3.145, 8.671) for the youngest age group. Using this fit to the infection of the youngest children, we then attempt to find what mechanisms of immunity can alter this infection curve to produce the curves observed in older age groups.

## Results

### Modelling the effects of liver stage immunity

Previous work suggests that immunity may act anywhere in the pre-erythrocytic stage to block sporozoite invasion [14] or kill infected liver cells. Wherever it acts, the primary results of pre-erythrocytic immunity will be to (i) reduce the proportion of infected bites that lead to blood stage infection (reduce *k*), and/or (ii) to reduce the number of infected liver cells that successfully mature to release merozoites and initiate the blood stage (reduce *A*).

**Figure 2:**
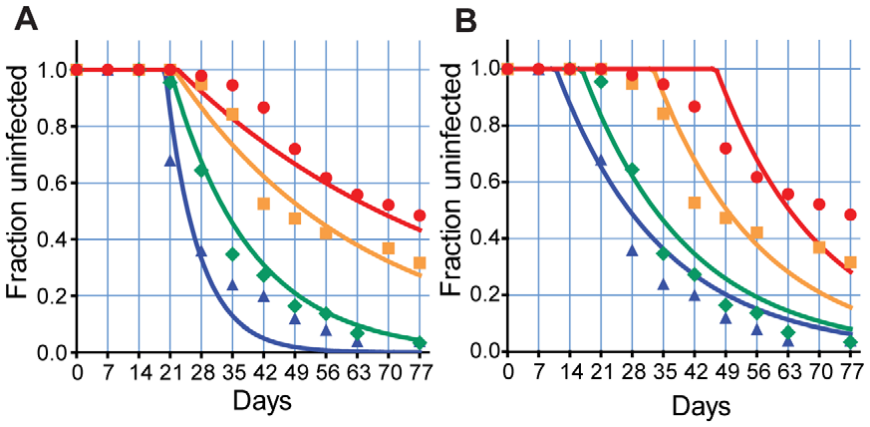
The impact of immunity on reinfection curves. The effects of liver stage immunity blocking a proportion of infected bites (A), or blood stage immunity decreasing the growth of parasites (decreased PMR) (B) is shown. Liver stage immunity alone decreases the rate of new infections, producing a family of exponential curves. Slower growth produces a family of curves shifted due to an increasing delay in detection. The same biting rate is used for all age groups. Blue triangle and blue line- C1, green diamond and green line - C2, orange square and orange line - C3, red circle and red line - A.

From a modeling perspective, anything that reduces the initiation of successful blood stage infection has the same effect – it simply decreases the slope of our reinfection curve (which remains in exponential form). Fig. 2.A. illustrates the predicted effects of decreasing the rate of initiation of blood stage infection and shows the best fits of a model that incorporates this change to the cohort data. The fits are essentially a family of curves commencing at the same time, and decreasing at different rates. The best fit parameters and goodness of fit statistics are in the [Text S4], (model 1). This is quite different from the observed infection rates in two respects: (a) it does not capture the greater delay until infection is observed in older children and adults, and (b) it does not capture the convex part of the adults' and older children's infection curve.

One may think that greater delay in detection of blood stage infection in adults may be explained by other factors such as increased blood volume in adults, or a decrease in the number of infected liver cells surviving to maturity in adults (due to the effects of cytotoxic T cell immunity killing [13] or growth inhibition [15]). However, this is not adequate to explain the observed delay. The number of infected liver cells is rarely higher then 10–14, [13], [16], [17], and the blood volume differs by only a factor of approximately 5, thus even combined they could explain at best a delay of 4.7 days (using the PMR of the youngest age group 5.9). It is clear that, in addition to underestimating the delay, pre-erythrocytic immunity alone fails to account for the change in shape of the curve associated with age and cumulative exposure.

### The effects of blood stage immunity

Once the blood stage of infection is successfully initiated, immunity may act on either the growth of the parasite within the infected RBC or directly on the released merozoites to reduce the rate of successful subsequent invasion events. Immunity acting at these stages will have the effect of reducing the parasite multiplication rate, and reduced PMR will lead to a greater delay until infection is detected. However, reducing the PMR alone affects the infection curves simply by shifting the curves to the right. That is, if the rate of successful initiation of the liver stage is unaffected, the effect is only to change the time until parasite density grows above the threshold but not to affect the infection rate.

We attempted to fit our model assuming only the PMR changed with age (Fig. 2.B). The best fit parameters and goodness of fit statistics are in the [Text S4], (model 3).. It is once again clear that although growth-inhibiting immunity can account for the delay in infection, it fails to capture the shape of the infection curve. Even combining the effects of liver and blood stage immunity fails to capture the observed infection curves. For example, in the adult infection curves, no combination of liver and blood stage immunity can account for the delayed, slowly starting shoulder of the infection curve, followed by an early period of high infection, followed by a further slowing in infection rate (Fig. 1).

### The effects of blood-stage immunity with a distribution in parasite growth rates

The simple models above consider the effects of a fixed growth rate within a given age group. However, the growth rate of parasites is likely to vary between individuals as a result of a combination of parasite strain and host factors such as intrinsic immune maturation [18], [19]. Therefore, rather than consider all infections in an age group having the same growth rate [PMR = 6, for example], we investigated the effects of variation in the parasite growth rate around the population mean within each group. For simplicity we have assumed a normal distribution in PMR, and the same standard deviation (as a proportion of the mean) for each age group. We note that it is not essential that all groups have the same coefficient of variation, however fitting the same parameter to all reduces the number of parameters fitted by three (See [Text S1] for a complete description of the mathematical model).

Using a simple assumption that all age groups have the same biting rate and the same type of positive normal distribution of growth rates (with the standard deviation the same proportion of the mean PMR) we then fitted a theoretical infection function (2) to the observed infection proportion, allowing only the mean PMR to vary between age groups (note that a higher biting rate in adults has been suggested by some [20] and can also be accommodated in the model).
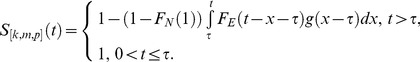
(2)
*g* (.) is the probability density function of the delay to detection,
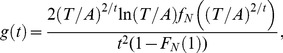
(3)
*f_N_*_(*μ,pμ*)_ (*x*) is the probability density function of a normal distribution with parameters *μ* and *pμ*, and *μ* is the mean PMR in a given age group. Constant *p* is a positive number same for all age groups. *F_E(k)_*(*x*) is the cumulative density function of an exponential distribution with parameter *k* – the average number of bites per day, which is also the same for all age groups.

The derivation of the theoretical infection function that incorporates distribution in the time to infective bites and PMR within the host for different age groups is shown in [Text S1]. The best fit parameters and goodness of fit statistics are in the [Text S4], (model 4).

**Figure 3:**
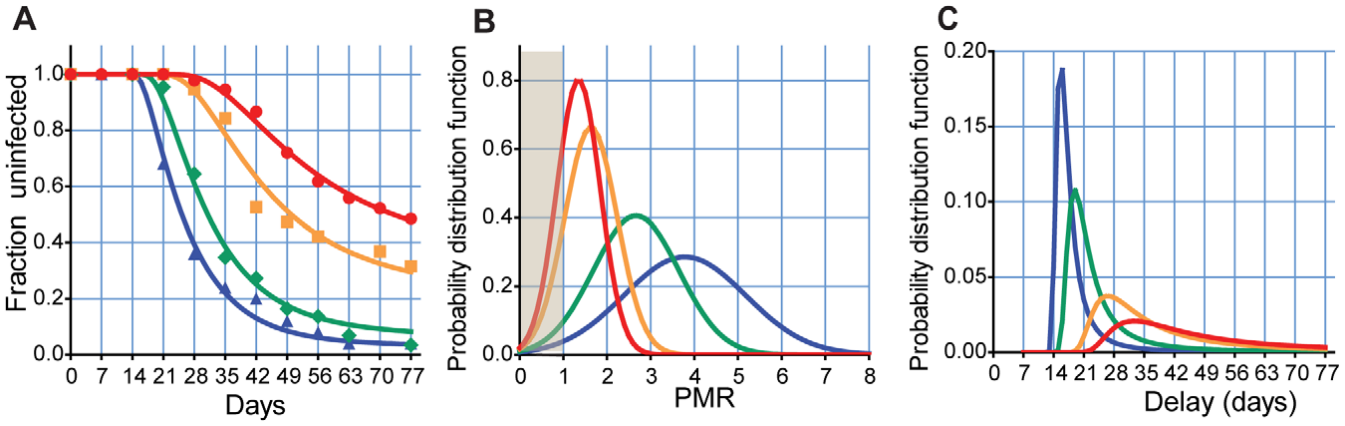
A distribution in growth rates explains differences in reinfection curves. Panel A. Optimal fit of a model allowing variation only in mean parasite multiplication rate (PMR) between groups. Panel B. The mean PMR for each group and the distributions. The shaded area at left indicates where PMR <1, and the parasite population does not grow, Panel C. Given the distribution in PMR, we can also calculate the distribution of delay as a distribution of a function of a random variable. Blue triangle and blue line- C1, green diamond and green line - C2, orange square and orange line - C3, red circle and red line - A.

Remarkably, allowing only the average PMR to vary between age groups captures the main features of the natural infection profiles. We see both the increasing delay with age, as well as the early rapid increase in infection rates followed by an apparent slowing down of the rate of infection (Fig. 3A). With this model of a distribution in PMRs, we can now understand the unusual shape of the adult infection curve: The early slow shoulder of the curve represents the small fraction of infections that grow rapidly, and are detected early (the right hand side of the distribution in Fig. 3B). The rapid phase of infection is around the mean of the PMR curve, and the apparent slowing represents the very slowly growing infections, which are not detected during the 11 weeks of analysis. Because of the distribution of growth rates, there is a proportion of infections where the PMR <1 (shaded in Fig. 3B), implying the number of parasites decreases at each round of infection (each currently infected RBC infects less than one RBC in next round). In children, with mean PMR of ≈3.8, only a small proportion of infections have PMR <1. However, for the adults, with mean PMR ≈1.35, a large proportion of bites (≈24%) have PMR <1, which is why we see an apparent slowing of the infection rate later in the study. We note that by contrast simply allowing a distribution of the level of liver stage immunity alone does not improve the fit of the liver stage model (see [Text S3] for a detailed description of the model ).

### Modelling naturally acquired immunity

Our analysis suggests that a model that can fit the data well is one that assumes a distribution in PMRs within each age group, and a decrease in mean PMRs with age. A key question that follows is – could such a distribution with age arise from a known mechanism of immunity? Similarly, we have considered above only the relationship between PMR and time to detection of infection. However, decreased PMR would also decrease the observed levels of parasitaemia with age. Is the change in PMR required to produce the reinfection curves consistent with the change in parasitaemia levels with age? To answer this, we developed a stochastic model of within-host immunity and parasite growth to explore the effects of naturally acquired immunity. We focused on the impact of immunity affecting parasite growth, and further allowed that such immunity might either be strain-specific, or ‘general’ (affecting all strains equally). In this context, a strain-specific immunity may be defined by expression of variable proteins such as var genes or immunologic targets proteins such as merozoite or sporozoite surface antigens) that can vary across strains. General immunity is a response (be it immune or physiological – such as hyper-splenism) that acts equally on all strains. A number of previous models of malaria immunity have considered the effects of partially cross-reactive immune responses [21], [22], [23], [24]. However, we effectively model only the extremes of ‘completely strain-specific’ or ‘completely cross reactive’ responses for simplicity.

In our model, strain-specific immunity arises as the result of the infection with a certain strain and neutralises the parasites of this strain only. General immunity arises as the result of infection with any strain and it can neutralise parasites of any strain. The acquisition rate of both types of immunity is proportional to concentration of parasites in the blood. Without infection, the existing level of immunity decays at some constant rate. Thus the model has four parameters of immunity: for both strain-specific immunity and general immunity we have a rate of increase in immunity per unit parasite (denoted by *α* and *γ* respectively), and a rate of loss of immunity in the absence of parasite denoted (denoted by *β* and *δ* respectively). We also require three parasite parameters: the mean number of bites per day *k* (biting rate), the baseline multiplication rate *r* of the parasites, and the number of different strains *n*. The basic equations of parasite – immunity dynamics of the model are:
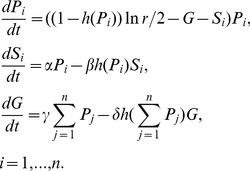
(4)Here *P_i_* is the concentration of parasites of the strain *i*, *S_i_* is the strength of the *i* ^th^ strain specific immunity, 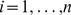. *G* is the strength of the general immunity. The function *h(.)* is either one (if the concentration of parasites<threshold) or zero (if the concentration of parasites≥threshold), and allows for the elimination of parasites and the decay of immunity when the level of parasites drops below a threshold *Z* (in this case 0.005 parasites/microlitre ).

A detailed description of the mathematical model is in the [Text S2].

Using this model we can simulate the ‘life history’ of an individual in a malaria endemic area. In the model, we start with the assumption that all individuals are identical and lack immunity at birth (*t* = 0), and are then exposed to bites from different strains arriving at random times, with a random order of strains (choosing from our 50 notional strains), to which they then develop immunity (ignoring the transient contribution of maternally-derived immunity). Thus, the timing of bites and which strain is inoculated is stochastic, but between bites the dynamics of parasites and immunity is deterministic (as in equation 2).

**Figure 4:**
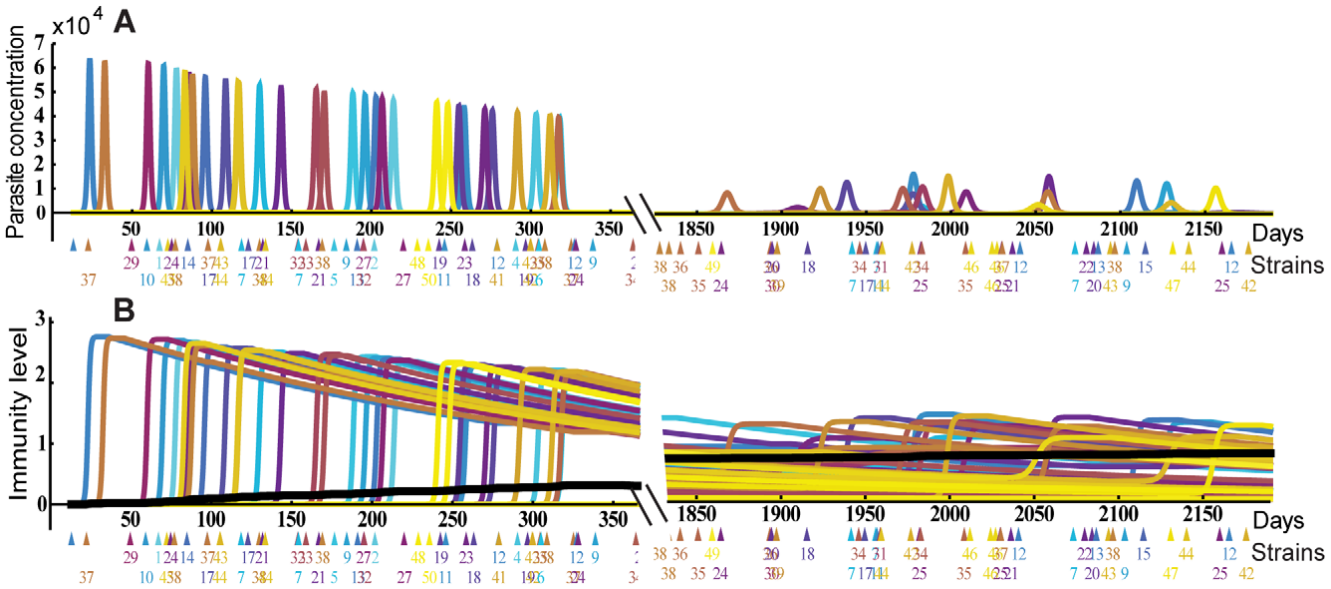
Evolution of immunity over time. Panel A. The results of a stochastic simulation of acquired immunity are shown for one *in silico* individual. Bites (coloured triangles) occur randomly with a fixed biting rate, and different strains (coloured, and numbered below) initiate infection. Each infection induces and is cleared by strain-specific immunity (Panel B), which then wanes over time. General immunity (solid black line) accumulates slowly over time. The accumulation of immunity in the first year (left panels) and aged 5–6 years (right panels) is shown.

**Figure 5:**
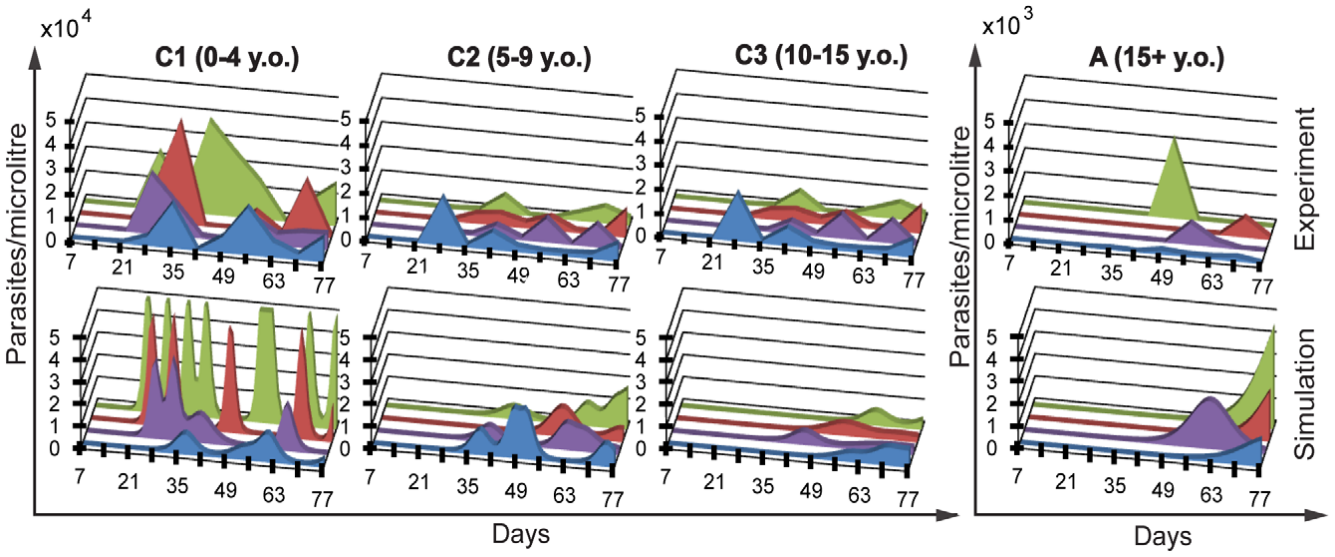
Modeling the dynamics of reinfection. The parasitaemia during reinfection in four individuals from the field study (top row) and in four individuals in the simulation (bottom row) are shown. Each individual is shown as a different colour.

**Figure 6:**
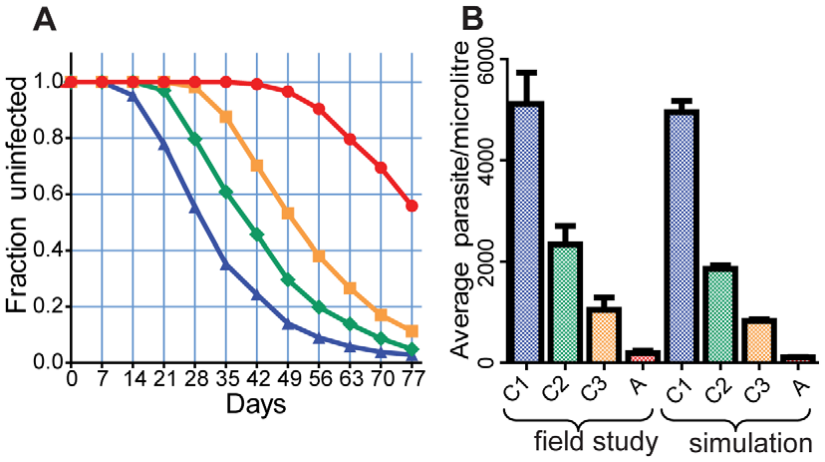
Reinfection and parasitaemia with blood stage immunity. The dynamics of reinfection (A) and the mean parasitaemia (B) are shown for the stochastic simulation of acquired blood stage immunity. Panel A. Mean (calculated every 7 days) of 5 simulations of the reinfection curves of individuals with general and strain specific immunity with parameters listed in Table S2.1. in [Text S2]. Blue triangle and blue line- 0–4 y.o., green diamond and green line - 5–9 y.o., orange square and orange line - 10–14 y.o., red circle and red line - 15–30 y.o. Panel B. Average concentration of parasites in blood in the field study data (left 4 bars) and in the simulated reinfection data presented in the panel A.

Fig. 4 shows the dynamics of parasite infection and acquisition of strain specific and general immunity by an individual, starting from birth to one year [left panel] and five to six years [right panel]. The top panels show the dynamics of parasite infection, as different parasite strains (indicated by different colours) initiate blood stage infection, grow, and then induce strain-specific immunity, leading to their clearance. Given the long half-life of strain-specific immunity and the absence of within-host parasite antigen variation in our model, each new parasitemia peak represents infection with a new strain. In addition to inducing strain-specific immunity (bottom panels, coloured), these infections also induce general immunity (solid black line), which accumulates over time. By simulating the life history of a small population of individuals (n = 50), we can then apply the concept of ‘treatment-time-to-infection’ trials to the simulated individuals. That is, by removing all blood stage parasites of individuals in different age groups and observing the time until parasite levels reach our detection threshold, we can simulate (re)-infection (Fig. 5 and 6). Fig. 5 shows the dynamics of parasitaemia during (re)-infection from the field study data (top) and the simulation (bottom), for four subjects from each of the different age groups. Remarkably, the simulation captures a number of the factors observed in our observational study; firstly, the natural infection curves show increasing delay with age and an increasing proportion of individuals remaining uninfected. Secondly, the observed reduction in parasite levels in blood with age is also captured in the model, indicating that the decreased PMR required to produce the reinfection curves is consistent with the decreased PMR required to produce the observed reduction in parasitaemia with age. (as higher immunity means parasites are controlled at a lower parasitaemia) (Fig. 5). Fig. 6A shows the infection curves for the whole simulated population, and Fig. 6B compares the mean parasitaemia for the field study data and simulation for different ages. Importantly, the major factor that can account for both delayed infection and lower parasitaemia in adults is simply a reduced average growth rate of parasites with age and naturally acquired immunity.

## Discussion

### Understanding the dynamics of immunity to malaria

A number of modelling studies have previously been applied to understanding the dynamics of malaria infection and the impact of immunity. Some of these studies have focused upon the dynamics of experimental infection of neurosyphilis patients in the USA during the early 20^th^ century with both *P. falciparum* [25] and *P. vivax* [26]. Such infections with *P. falciparum* often show a dynamic of repeated recrudescence, in the absence of reinfection. Modelling both infection rates and infection dynamics has also been applied to more recent data on infection in naturally exposed individuals [27], [28]. The recent discovery of the molecular mechanisms of antigenic variation in *P.falciparum* has also driven studies modelling the dynamics of immune interaction with an antigenically variable pathogen [21], [22], [23], [24]. In our study, we utilise field data on malaria infection dynamics after a short treatment course in a holoendemic region of Kenya. The estimated P. falciparum infection rates are extremely high, especially amongst young children, but consistent with the high entomological infection rates measured in this area [29]and with the blood-stage infection rates previously observed by others in similar studies [30], [31]. The prior treatment of patients gives an ideal opportunity to study malaria incidence in the absence of recrudesence from prior infection. However, there is a potential problem equating the measured time to reinfection in treated, asymptomatic individuals with incidence rates of new blood-stage infection in individuals living in highly endemic areas, and then using these findings to make general statements about acquired immunity to malaria. However, given that children in this area are treated on average twice a year [32], this is likely as natural an infection dynamic as can be readily studied.

Fitting of a simple model of infection dynamics to the field data on *P. falciparum* infection demonstrates that the observed rates of infection with age are consistent with a form of immunity that reduces the PMR of the parasite in blood. This mechanism of blood-stage immunity reducing PMRs is consistent not only with the dynamics of infection after treatment in different age groups, but also with the observed reduction in peak parasitaemia with older age groups (Fig. 6). Although clinical malaria was not studied in this model, one might imagine a reduction in parasite replication with age may also play some role in both the observed reduction in episodes of severe malaria as well as an apparent shift from acute to chronic infections with age. The fact that the model fits well without invoking a need for liver-stage immunity does not exclude a role for liver stage immunity, but suggests it is not a major force shaping the observed infection dynamics. By contrast, liver-stage immunity alone would be expected to have relatively little impact on either the delay or peak parasitaemia. The estimated PMR in this study and their change with age are very consistent with published studies of parasite growth in unexposed adults in the UK (estimated to have a high PMR of approx 8–14/cycle [12], [13]), compared with the reported very low PMRs in malaria endemic areas (1.6–3/cycle) [33], [34].

### Insights into specific and general immunity

Although the initial model was developed on the basis of a number of heuristic assumptions, the model provides some useful insights into the possible mechanisms of blood-stage immunity induced from natural malaria infections. Firstly, ‘parasite clearing’ immunity must be highly strain-specific: That is, we often see one parasite peak being cleared immediately before another peak arises (Fig. 5, C1, top). Given the time taken for clearance and growth, this means that we must have one parasite being cleared while another grows. Thus the immune response mediating clearance of individual parasite peaks must have the characteristics of being both highly strain-specific, and rapidly induced. Although this strain-specific response must be rapidly induced, the duration of strain-specific immunity may be varied in the model, depending on the number of strains used and the biting rate. That is, if there are only a small number of immunologically distinct strains of parasite, then the duration of immunity needs to be short – to allow reinfection. However, if there are a large number of immunologically distinct strains, the half-life of strain-specific immunity could be considerably longer and still consistent with the data. Thus we are not able to speculate on the relative duration of strain-specific versus general immunity, as has been discussed elsewhere [21], [22], [23], [24].

The long timescale over which age-related immunity is acquired suggests that whatever factor mediates this must have a long half-life (since the time taken for the immunity level to reach its maximum is related to the half-life of immunity). If specific immunity were the only mechanism, then it would need to be long-lived, and the age-associated acquisition of immunity would simply be the progress from having immunity to a few strains, to having immunity to most strains. However, the intermediate phenotype here (having immunity to a proportion of strains) is incompatible with the age and parasitaemia data. That is, if we had immunity to 50% of strains, and no immunity to the other 50%, this would mean that 50% of bites would be blocked (changing the reinfection curve as in Fig. 2A) and that parasitaemia of the remaining strains would be ‘normal’. This is clearly not consistent with the data, suggesting that although strain-specific immunity is required to explain the clearance of individual parasite peaks, it is not sufficient to explain age-associated changes in reinfection and parasitaemia.

The alternative to a strain-specific immune response to individual infection episodes is a general anti-parasite response that decreases the growth of all strains. In contrast to specific immunity, general immunity takes many years to acquire and accumulates after repeated infections. Thus, each infection should both induce only a small rise in general immunity, and it must be long-lived. It is also clear that general immunity alone cannot produce the observed infection dynamics, as this could not simultaneously drive one parasite strain down while the other grows. In broad terms, we might consider that an increase in general immunity with time drives down the mean PMR of an age group. The distribution in growth rates within an age group arises due to strain-specific immunity, with individual strains (in different individuals) having different growth rates due to the variable levels of strain-specific immunity, which wanes over time after infection with that strain.

### Mechanisms of immunity

Clinical studies of immune correlates of protection from malaria are often complicated and difficult to reconcile (reviewed in [8]). Overall, antibody levels have been found to generally increase with age and exposure, when measured by the ability to bind parasite antigens in ELISA. It has generally been difficult to demonstrate a clear correlation with protection from parasitaemia, as this is so strongly confounded by age. These antibody levels are also long-lived, appearing to persist with a half-life up to 5–10 years [35]. However, it is difficult to differentiate between binding antibodies exerting a direct protective effect, or merely acting as biomarkers of past infections.

Our model does not address the particular mechanisms or specificity of immunity. However, one prediction of the model is that each patent infection is cleared by a highly strain-specific response, as it is common that the clearance of one strain occurs while infection with another strain is still growing. This is consistent with early studies suggesting that growth-inhibitory antibodies may be highly strain-specific when tested in clinical isolates [36]. Interestingly, some growth-inhibitory antibodies appear as a correlate of protection within an age group, but paradoxically decrease with age [3], [10]. It is interesting to note that strain-specific immunity also paradoxically decreases with age in our model, due to the increase in general immunity.

We note some difficulties with defining the term ‘strain’ in malaria, as recent deep sequencing studies have suggested a wide range of genotypes [37]. Moreover, even once the genetic structure of malaria strains is resolved, the immunological specificity and cross-reactivity of responses may be highly complex. We do not use the term ‘cross-reactive immunity’ when describing a factor that limits the growth of all strains, but instead prefer the term ‘general immunity’. The concept of cross reactivity may tend to imply specific antibodies that cross-react between different strains. However, we do not wish to exclude other factors such as an enlarged spleen may tend to decrease the PMR of all strains, and may also play a role in protection.

The majority of cases of severe malaria occur amongst young children in endemic areas, and age and persistent exposure provide some level of immunity to both clinical symptoms, as well as measured parasitaemia [38]. Although this age-associated acquisition of immunity is known, clinical studies of immune responses to malaria often struggle to identify the mechanisms of protection [38]. We have employed an alternative strategy in an attempt to deconstruct the mechanisms of naturally acquired immunity based on the dynamics of infection in otherwise healthy asymptomatic individuals. We find that simple pre-erythrocytic immunity acting alone is inconsistent with the data, as is a highly strain-specific form of blood-stage immunity, as both would have the overall effect of merely decreasing the number of successful infections. Instead, the observed dynamics of infection and parasitaemia levels in a cohort of individuals from a holoendemic area of western Kenya are consistent with the reduction in parasite multiplication rate with age, leading to both a delay in the time until infection is detected, as well as reduce the peak parasitaemia. In terms of the clinical outcomes observed, our study suggests that the reduced rate of clinical malaria and reduced rate of apparent infection (measured at a particular threshold of parasitaemia detection) may both be driven by a reduction in parasite growth rate, independent of any real change in infection rate. Further studies are required to deconvolute immunity that inhibits parasite growth distinct from its ability to infect hepatocytes and erythrocytes.

## Supporting Information

Text S1 **Deterministic modelling of the effects of PMR.**
(PDF)

Text S2 **Stochastic modelling of blood stage acquired immunity.**
(PDF)

Text S3 **Liver stage immunity model (with the distribution of the strength of liver stage immunity).**
(PDF)

Text S4 **The best fit parameters and goodness of fit statistics for different models.**
(PDF)
