# Supplementary material for: The Dynamics of Naturally Acquired Immunity to Plasmodium falciparum Infection
Source: PLoS Comput Biol. 2012 Oct 18;8(10):e1002729. doi: 10.1371/journal.pcbi.1002729 (PMC3475668; doi:10.1371/journal.pcbi.1002729)
Supplement: Text S1 — Deterministic modelling of the effects of PMR. (PDF) [file pcbi.1002729.s001.pdf]

SUPPORTING TEXT S1

Deterministic modelling of the effects of PMR.

After determining that simply changing the (fixed) biting rate or PMR did not provide good fits to the infection curves, we set out to explore the effects of population variability in these parameters.

We assumed that the parasite multiplication rate (PMR) has a normal distribution within each age group *i* with the mean μ_i and the standard deviation $\sigma_i = p\mu_i$ ($i = 1,\ldots,4$) proportional to the mean, where *p* is a positive number and is the same in all groups. A PMR <1 implies that for each currently infected RBC, less than one newly infected RBC will be produced in the next round of infection – so the parasite will not grow. The following mathematical model describes the exponential growth of parasites during the early blood stage.

Let $C(t)$ denote the concentration of parasites at a time *t* after emerging from the liver. It is easy to see that

$$C(t) = Ar^{t/2}, \qquad (S1.1)$$

where *r* is the average PMR, and constant *A* is the concentration of parasites in blood at the beginning of the blood stage .

Let us denote the probability density function (PDF) and cumulative density function (CDF) of the normal distribution by $f_N(.)$ and $F_N(.)$ respectively. Knowing the PDF of the PMR and the relation between PMR and the delay to detection, we can find $g(t)$- the PDF of the delay, for the fraction of population of $1-F_N(x)$ which has the PMR >1. To do this we used the formula for the distribution of function of random variable. It requires the inverse function to the delay function of *r*. The inverse function $r(t) = t(r)^{-1} = (T/A)^{2/t}$, was found from equation

$$C(t) = T, \qquad (S1.2)$$

its first derivative is $r'(t) = 2(T/A)^{2/t}\ln(T/A)/t^2$. Thus the distribution function of the delay will have following form

$$g(t) = \frac{f_N(r(t))|r'(t)|}{1-F_N(1)} = \frac{2(T/A)^{2/t}\ln(T/A)\, f_N\left((T/A)^{2/t}\right)}{t^2(1-F_N(1))}, \qquad (S1.3)$$

Let us introduce a time constant τ as the earliest possible moment of blood stage infection in our model. It means that all blood stage infections that had been present in the blood before the τ *-th* day were killed by the anti-malaria drug.

Because infective mosquito bites occur continuously and independently of each other, we are to expect that the waiting time until an infective bite and therefore the time until successful initiation of blood stage will have an exponential distribution, and we can assume that the average number of successful initiations of blood stage infections per day is equal to k. Let us denote the CDF of an exponential distribution with parameter k by $F_E(.)$. Now we can find the infection function $S(t)$, which includes the convolution of the CDF of the exponential distribution with variable initial plateau (delay to detection) and the distribution of the delays. Parameters of the model are in the square brackets.

$$S_{[k,m,p]}(t)=\begin{cases}1-\left(1-F_N(1)\right)\int_{\tau}^{t}F_E\left(t-x-\tau\right)g\left(x-\tau\right)dx,\ t>\tau,\\ 1,\ 0<t\le\tau.\end{cases}\qquad (S1.4)$$

By denoting, $\hat{g}(t)=(1-F_N(1))g(t)$ we obtain a shorter expression for the infection function:

$$S_{[k,m,p]}(t)=\begin{cases}1-\int_{\tau}^{t}F_E\left(t-x-\tau\right)\hat{g}\left(x-\tau\right)dx,\ t>\tau,\\ 1,\quad 0<t\le\tau.\end{cases}\qquad (S1.5)$$

In our model k and p are shared parameter between groups and m is different for each group (we denote them by m_i). Thus, we must fit only 6 parameters. For fitting, we used NonlinearModelFit function in Wolfram Mathematica®, Wolfram Research, Inc, Champaign, IL,

The values of all constants in the model were taken from previously published papers.

To estimate the initial concentration of parasites in i-th age group A_i,we need to know the number of initially infected RBC and the average blood volume in the age group. The average blood volumes in age groups V_i, i=1,...,4, were found from the Chart 1 in reference [1] that gave us the values V_1 =1.1x10^6µl, V_2 =2 x10^6µl, V_3 =3.3 x10^6µl, V_4 =5 x10^6µl. The initial number of parasitized RBC for a single bite was estimated as 5.6x10^4 knowing that after 5 simultaneous bites the initial number of infected RBC was 28*10^4 [2]. The first possible moment of blood stage infection τ was taken as equal to 7 days, since the blood concentration of lumefantrin even by day 7 post treatment is high enough (more than 280 ng/µl) to kill a relatively small number of parasites released from liver (reported in [3,4]). The detection threshold T was taken as equal to

40 parasites per microlitre, since this was the minimal concentration in the analysed data table which completely conforms to the range of 20-50 parasites/microlitre reported in [2,5]. However, the variation of the above-mentioned constants does not significantly affect the optimal values of PMR. This is easy to see from the relation $r = (T / A)^{2/t}$ where t is the time between the beginning of the blood stage and the detection of parasitaemia. We observe that t is more than 6 days. Consequently, the exponent $2/t<1/3$ and the variation of the constant *T/A* will not significantly affect the PMR.

The best fit parameters are in the [Text S.4].

1. Seckei H (1936) Blood volume and circulation time in children. Archives of Disease in Childhood 11: 21-30.
2. Bejon P, Andrews L, Andersen RF, Dunachie S, Webster D, et al. (2005) Calculation of liver-to-blood inocula, parasite growth rates, and preerythrocytic vaccine efficacy, from serial quantitative polymerase chain reaction studies of volunteers challenged with malaria sporozoites. Journal of Infectious Diseases 191: 619-626.
3. Ezzet F, van Vugt M, Nosten F, Looareesuwan S, White NJ (2000) Pharmacokinetics and pharmacodynamics of lumefantrine (benflumetol) in acute falciparum malaria. Antimicrobial Agents and Chemotherapy 44: 697-704.
4. White NJ, van Vugt M, Ezzet F (1999) Clinical pharmacokinetics and pharmacodynamics of artemether-lumefantrine. Clinical Pharmacokinetics 37: 105-125.
5. Andrews L, Andersen RF, Webster D, Dunachie S, Walther RM, et al. (2005) Quantitative real-time polymerase chain reaction for malaria diagnosis and its use in malaria vaccine clinical trials. American Journal of Tropical Medicine and Hygiene 73: 191-198.
