# Supplementary material for: The Dynamics of Naturally Acquired Immunity to Plasmodium falciparum Infection
Source: PLoS Comput Biol. 2012 Oct 18;8(10):e1002729. doi: 10.1371/journal.pcbi.1002729 (PMC3475668; doi:10.1371/journal.pcbi.1002729)
Supplement: Text S2 — Stochastic modelling of blood stage acquired immunity. (PDF) [file pcbi.1002729.s002.pdf]

SUPPORTING TEXT S2

Stochastic modelling of blood stage acquired immunity

In order to explore the effects of acquisition of immunity with age, we developed a stochastic model in which we tracked over time a number of individuals, who varied in the timing and strain of the malaria infections they encountered. Once infection was initiated by a particular strain, the intrahost dynamics and acquisition of immunity were deterministic (until the next random bite).

The system of ODEs (S2.1) describes the acquisition of general and strain specific immunity to malaria by an individual during the time period between infectious bites.

$$\begin{aligned}
&\frac{dP_i}{dt} = ((1-h(P_i))\ln r/2 - G - S_i)P_i,\\
&\frac{dS_i}{dt} = \alpha P_i - \beta h(P_i)S_i,\\
&\frac{dG}{dt} = \gamma\sum_{j=1}^{n} P_j - \delta h(\sum_{j=1}^{n} P_j)G,\\
&P_i(t_0) = P_{i0},\ S_i(t_0) = S_{i0},\ G(t_0) = G_0,\ i = 1,\ldots,n.\\
&h(x) = \begin{cases} 1, x < Z,\\ 0, x \ge Z.\end{cases}
\end{aligned} \qquad (S2.1)$$

Here P_i is the concentration of parasites of the strain i, S_i is the strength of ith strain specific immunity, $i = 1,\ldots n$, n is the maximal number of strains, G is the strength of the general immunity, and r is the PMR in a malaria naïve person (when G and S_i for all strains i=1,...,n, are equal to zero). Immunity stimulation coefficients for strain specific and general immunities are α and γ respectively. Rates of loss of strain specific and general immunity are β and δ. We assumed that the loss of immunity occurs only when concentration of parasite drops below some small number Z.

Initial values are determined by the following 3 rules:

1) Initial values for all variables for current period are equal to values of the corresponding variable at the end of previous period or 0 if the current interval is the first one;

2) If after application of rule 1 the initial concentration of parasites P_{i0} for some strain i is less than Z, then P_{i0} =0, because otherwise the parasite would never vanish;

3) If the current bite inoculates parasites of strain i, then $P_{i0}=P_{i0}+\varepsilon/V(t)$, after the application of rule 1 and 2. The constant ε is the initial number of infected RBC, $V(t)$ is the blood volume at age t.

The function $V(t)$ was estimated to be the linear approximation of the Chart 1 in reference [1] and formally can be written as

$$V(t)=\begin{cases}0.00059t+0.3, t<22\times365(days),\\ 5, t\geq22\times365(days).\end{cases}$$

Infectious bites occur randomly and time between infectious bites has an exponential distribution with the mean rate k, as discussed in the [Text S1]. All strains are equally probable and have equal PMRs in a naive individual.

Meaning of the parameter	Symbol	Value
Number of individuals in every age group	N	50
Number of strains	n	50
General immun. acquisition rate	γ	$6*10^{-8}$
Initial PMR (per cycle)	r	16
General immun. loss rate	δ	0.005
St. specific immun. acquisition rate	α	$1.5*10^{-5}$
St. specific immun. loss rate	β	0.003
Average number of bites per day	k	0.11
Initial number of infected RBC (in millions)	ε	0.056
Detection threshold (parasites/µl)	T	40
Zero level (parasites/µl)	Z	0.005

Table S2.1. Baseline Parameters of simulation of infection presented in fig. S2.1.

The simulation program was written in Wolfram Mathematica®, Wolfram Research, Inc, Champaign, IL, and the system (S2.1) was solved using function DSolve.

Initially we used the simulation to create 50 individuals from 0 to 30 “years” old. The age groups were formed by selecting the values of variables S_i and G of each individual at the

random age within the required age range of the group. The selected values of S_i and G are then used as the starting values for simulation of infection.

The first phase of infection simulation is the treatment. It was modeled as a seven day period without parasites in blood and infective bites. It means that the level of all immunities dropped to the level that can be calculated as

$$(current\ level) \times \mathrm{Exp}(-7 \times (loss\ rate)) \qquad (S2.2)$$

After this period, the exposure to infective bites was modeled by parasite-immunity dynamics model based on the system (S2.1) until the end of the surveillance period of 77 days. We defined the moment of the detection of infection as the time point when the parasite concentration reached the detection threshold T.

Reduction of PMRs with age.

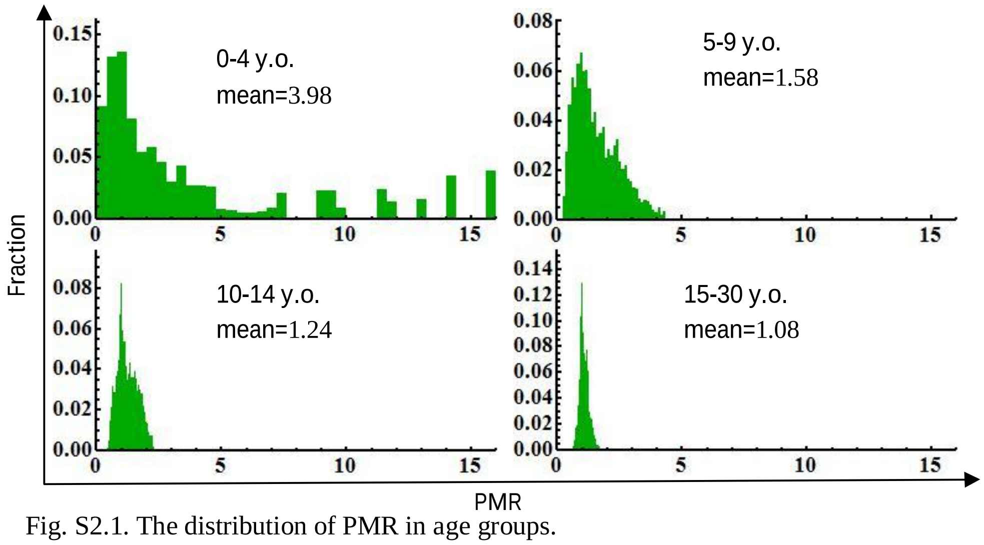

Fig. S2.1. The distribution of PMR in age groups.

Our stochastic simulations showed that the older groups have lower mean PMR at the beginning of infection, as it was predicted by fitting of the model (S1.5) in [Text S1]. From the 1st equation of the system (S2.1), PMR of the strain i at time t can be calculated as $PMR_i(t) = r\mathrm{Exp}(-2(S_i(t) + G(t)))$. The distributions of PMRs in Fig. S2.1. were estimated by the histograms of PMRs of the all strains in all individuals in age groups at the selection time.
