# Supplementary material for: The Dynamics of Naturally Acquired Immunity to Plasmodium falciparum Infection
Source: PLoS Comput Biol. 2012 Oct 18;8(10):e1002729. doi: 10.1371/journal.pcbi.1002729 (PMC3475668; doi:10.1371/journal.pcbi.1002729)
Supplement: Text S3 — Liver stage immunity model (with the distribution of the strength of liver stage immunity). (PDF) [file pcbi.1002729.s003.pdf]

**SUPPORTING TEXT S3**

**Liver stage immunity model (with the distribution of the strength of liver stage immunity).**

In the main text and [Text S1] we show that allowing a distribution of parasite growth (PMR) leads to a much better fit of the reinfection curve. Here, we demonstrate that allowing a distribution of liver stage immunity within age groups did not greatly improve the fit of the liver stage model. We assumed that the rate of initiation of blood stage infections has normal distribution with standard deviation proportional to the mean (*m*) in all groups, with the same proportionality constant (*p*). We assume the same PMR for all groups. The delay to detection as a function of PMR is $\theta(r) = 2\log_r T / A$ , can be found from equation S1.2 in [Text S1]. The definition and the values of all constants are the same as in the [Text S1].

Let us denote the probability density function of normal distribution and cumulative density function (CDF) of the normal distribution by $f_N(.)$ and $F_N(.)$ respectively. Then the infection function will be the integral of the exponential CDF with random parameter and the distribution function of this parameter. Since we do not integrate from minus infinity to infinity, we normalize the integral by dividing it by the fraction of values of the parameter $k$ in the interval between 0 and some maximal $k$ ($k_{\max}$).

$$S_{[k,r,\lambda]}(t) = \begin{cases} 1 - \int_0^{k_{\max}} (1 - e^{-k(t-\tau-\theta(r))}) f_{N(m,pm)}(k)\, dk \,/\, (F_{N(m,pm)}(k_{\max}) - F_{N(m,pm)}(0)), t > \tau, \\ 1, t \le \tau. \end{cases}$$

The model was fitted to each age group with $p$ and $r$ as shared parameters between groups and, $m$ different for each group (we denote them by $m_i$) using NonlinearModelFit function in Wolfram Mathematica®, Wolfram Research, Inc, Champaign, IL. The best fit parameters are in the [Text S4], model 2.

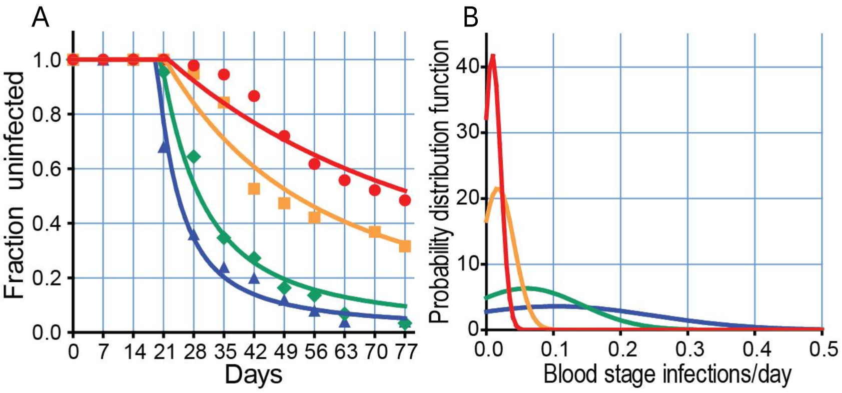


Fig. S3.1. A. The best fit of the model. B. Blue triangle and blue line- C1, green diamond and green line - C2, orange square and orange line - C3, red circle and red line - A.
