# Supplementary material for: The Dynamics of Naturally Acquired Immunity to Plasmodium falciparum Infection
Source: PLoS Comput Biol. 2012 Oct 18;8(10):e1002729. doi: 10.1371/journal.pcbi.1002729 (PMC3475668; doi:10.1371/journal.pcbi.1002729)
Supplement: Text S4 — The best fit parameters and goodness of fit statistics for different models. (PDF) [file pcbi.1002729.s004.pdf]

SUPPORTING TEXT S4

The best fit parameters and goodness of fit statistics for different models.

1.The model with the same PMR and different rate of initiation of blood stage infection.

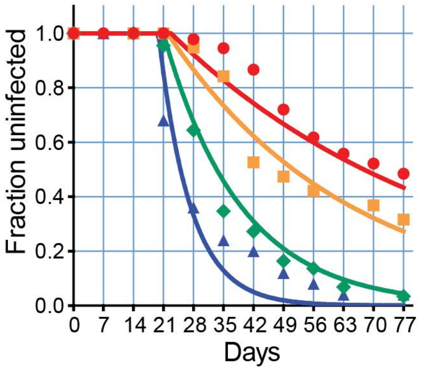

Blue triangle and blue line - C1, green diamond and green line - C2, orange square and orange line - C3, red circle and red line - A.

Parameter	Estimate	Units	95 % CI	
k_1	0.135	Blood inf/day	0.089	0.18
k_2	0.056	"	0.044	0.067
k_3	0.024	"	0.02	0.028
k_4	0.015	"	0.012	0.018
r	2.829	Inf . RBC /cycle	2.56	3.098
The goodness of fit statistics				
Residual Sum of Squares				0.18
Akaike information criterion				-90.55

2.The model with the distribution of successful biting rate.

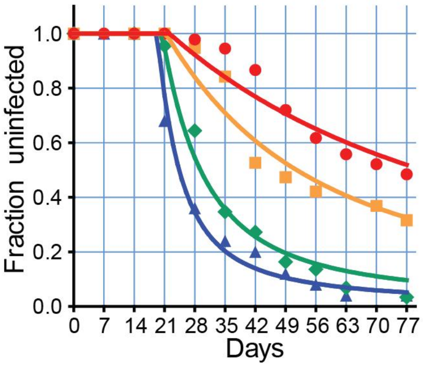

Blue triangle and blue line - C1, green diamond and green line - C2, orange square and orange line - C3, red circle and red line - A.

Parameter	Estimate	Units	95 % CI	
m_1	0.104	Blood inf./day	0.000	0.241
m_2	0.059	"	0.000	0.142
m_3	0.017	"	0.000	0.045
m_4	0.009	"	0.000	0.024
r	2.951	Inf . RBC /cycle	2.676	3.225
p	1.389		0.000	5.085
The goodness of fit statistics				
Residual Sum of Squares				0.106
Akaike information criterion				-109.7

3.The model with the same successful biting rate and different PMR.

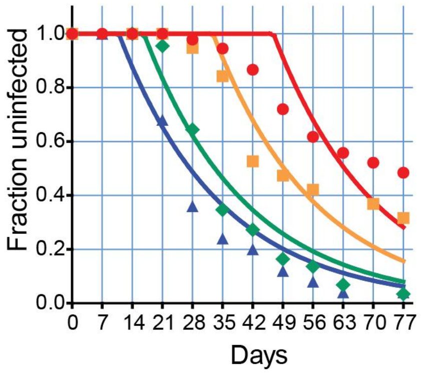

Blue triangle and blue line - C1, green diamond and green line - C2, orange square and orange line - C3, red circle and red line - A.

Parameter	Estimate		95 % CI	
r_1	32	Inf . RBC /cycle	0	153,5
r_2	4.48811	“	1.373	7.6027
r_3	1.82689	“	1.659	2
r_4	1.50842	“	1.447	1.57
k	0.0419	Blood inf./day	0.034	0.0495
The goodness of fit statistics				
Residual Sum of Squares				0.34
Akaike information criterion				-65.25

4. The model with the distribution of PMR.

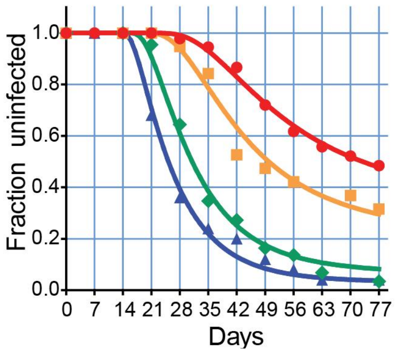

Blue triangle and blue line - C1, green diamond and green line - C2, orange square and orange line - C3, red circle and red line - A.

Parameter	Estimate		95 % CI	
m_1	3.785	Inf . RBC /cycle	2.724	4.846
m_2	2.667	“	2.256	3.078
m_3	1.632	“	1.537	1.728
m_4	1.349	“	1.291	1.406
k	0.109	Blood inf./day	0.072	0.146
p	0.369		0.327	0.412
The goodness of fit statistics				
Residual Sum of Squares				0.042
Akaike information criterion				-146.4
